# Circulating miR-99a-5p Expression in Plasma: A Potential Biomarker for Early Diagnosis of Breast Cancer

**DOI:** 10.3390/ijms21197427

**Published:** 2020-10-08

**Authors:** Iris Garrido-Cano, Vera Constâncio, Anna Adam-Artigues, Ana Lameirinhas, Soraya Simón, Belen Ortega, María Teresa Martínez, Cristina Hernando, Begoña Bermejo, Ana Lluch, Paula Lopes, Rui Henrique, Carmen Jerónimo, Juan Miguel Cejalvo, Pilar Eroles

**Affiliations:** 1 Biomedical Research Institute INCLIVA, 46010 Valencia, Spain; irgarca@doctor.upv.es (I.G.-C.); anna.adam.artigues@gmail.com (A.A.-A.); analameirinhas@gmail.com (A.L.); soraya_91_6@hotmail.com (S.S.); ortegamorillob@gmail.com (B.O.); maitemartinez3@yahoo.es (M.T.M.); c.hernandomelia@gmail.com (C.H.); begobermejo@gmail.com (B.B.); 2 Cancer Biology and Epigenetics Group–Research Center, Portuguese Oncology Institute of Porto (CI-IPOP), 4200-072 Porto, Portugal; vera.salvado.constancio@ipoporto.min-saude.pt (V.C.); ana.ambrosio@ipoporto.min-saude.pt (P.L.); rmhenrique@ipoporto.min-saude.pt (R.H.); 3 Clinical Oncology Department, Hospital Clínico Universitario de Valencia, 46010 Valencia, Spain; lluch_ana@gva.es; 4 Centro de Investigación Biomédica en Red de Cáncer (CIBERONC), 28029 Madrid, Spain; 5 Department of Medicine, Universitat de València, 46010 Valencia, Spain; 6 Department of Pathology, Portuguese Oncology Institute of Porto, 4200-072 Porto, Portugal; 7 Department of Pathology and Molecular Immunology, Institute of Biomedical Sciences Abel Salazar-University of Porto (ICBAS-UP), 4050-313 Porto, Portugal; 8 COST Action CA15204, 1210 Brussels, Belgium; 9 Department of Physiology, Universitat de València, 46010 Valencia, Spain

**Keywords:** breast cancer, biomarker, plasma, diagnosis

## Abstract

MicroRNAs have emerged as new diagnostic and therapeutic biomarkers for breast cancer. Herein, we analysed miR-99a-5p expression levels in primary tumours and plasma of breast cancer patients to evaluate its usefulness as a minimally invasive diagnostic biomarker. MiR-99a-5p expression levels were determined by quantitative real-time PCR in three independent cohorts of patients: (I) Discovery cohort: breast cancer tissues (*n* = 103) and healthy breast tissues (*n* = 26); (II) Testing cohort: plasma samples from 105 patients and 98 healthy donors; (III) Validation cohort: plasma samples from 89 patients and 85 healthy donors. Our results demonstrated that miR-99a-5p was significantly downregulated in breast cancer tissues compared to healthy breast tissues. Conversely, miR-99a-5p levels were significantly higher in breast cancer patients than in healthy controls in plasma samples from both testing and validation cohorts, and ROC curve analysis revealed that miR-99a-5p has good diagnostic potential even to detect early breast cancer. In conclusion, miR-99a-5p’s deregulated expression distinguished healthy patients from breast cancer patients in two different types of samples (tissues and plasma). Interestingly, expression levels in plasma were significantly lower in healthy controls than in early-stage breast cancer patients. Our findings suggest circulating miR-99a-5p as a novel promising non-invasive biomarker for breast cancer detection.

## 1. Introduction

Breast cancer (BC) is one of the most common malignant diseases in the world. In 2018, more than 2 million new cases were diagnosed, also being the leading cause of cancer-related death in women in over 100 countries [1]. Indeed, although a 100% 5-year survival rate is observed for BC patients diagnosed at stage I, it dramatically decreases to 26% for those diagnosed at stage IV [2,3,4]. Hence, new BC effective early-diagnosis methods are urgently needed to reduce its mortality rate. Currently, mammography is still considered the gold-standard method for the detection of BC. However, the sensitivity and specificity of mammography can be low in young women and women with dense breast tissue [5]. Additionally, because BC tumours are very heterogeneous, a tissue biopsy is mandatory to obtain the molecular classification of each tumour, that is based on the expression of several biomarkers, such as estrogen receptor (ER), progesterone receptor (PR), HER2 (human epidermal growth factor receptor 2) overexpression, or Ki-67, which determine the treatment choice [4,6,7].

Recently, microRNAs (miRNAs) have emerged as new diagnostic and therapeutic biomarkers for BC [3,8,9]. miRNAs are small non-coding RNAs of 19–25 nucleotides in length, which are key regulators of post-transcriptional gene expression through the silencing of messenger RNAs (mRNAs) by different mechanisms [10]. MiRNAs are involved in a wide variety of biological processes, such as proliferation, apoptosis, or cell cycle [9,11,12,13]. Therefore, the dysregulation of miRNA expression has been shown to have important effects on several diseases, such as autoimmune disorders, bone diseases, or cancer. Recently, due to their ability to regulate tumour initiation, progression, and metastasis, miRNAs have become promising BC biomarkers [3,8].

Indeed, several miRNAs were shown to be differentially expressed in breast tumours and healthy counterparts, and their expression levels have been related to immunohistochemical profiles, prognosis, response to treatment, or clinical outcomes [8,14].

MiRNAs are detectable in biological fluids that can be collected with minimal invasive techniques. Thus, the diagnostic and prognostic value of circulating miRNAs might be of great interest [9]. Several studies have been assessing the potential use of circulating miRNAs in plasma as biomarkers for different types of cancers and, nowadays, they are considered promising markers for diagnosis, prognosis, and treatment response [7,15,16,17,18]. In BC, it has been proven that circulating miRNAs differ between cancer patients and healthy volunteers [9,19,20]. Therefore, miRNAs could be used as effective minimally invasive biomarkers for the diagnosis and monitoring of BC patients. 

Mir-99a-5p, which functions as a tumour suppressor gene by inhibiting proliferation, migration, and invasion, has been found dysregulated in several tumours [21,22]. Specifically in BC tissues, miR-99a-5p has been consistently reported to be downregulated [23,24,25,26]. Hence, herein we sought to investigate the applicability of miR-99a-5p expression levels as a minimally invasive BC diagnostic biomarker.

## 2. Results

### 2.1. Study Design to Develop a Novel miRNA Biomarker

**Figure 1:**
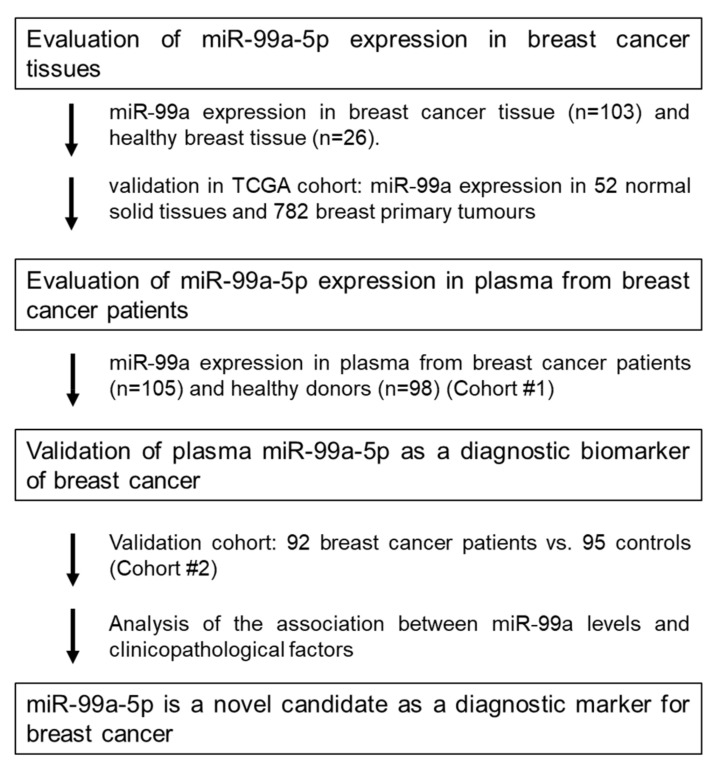
Study design to develop a novel miRNA biomarker.

Herein, we tested miR-99a-5p, as a candidate biomarker to diagnose BC. This study was divided into three parts: (1) Assessment of miR-99a-5p expression levels in BC and healthy breast tissue; (2) Evaluation of the miR-99a-5p expression levels in plasma of BC patients and healthy controls; (3) Validation of miR-99a-5p expression levels as a diagnostic biomarker for BC (Figure 1).

### 2.2. MiR-99a-5p Expression in Tissue

#### Cohort #1: Discovery Cohort

**Figure 2:**
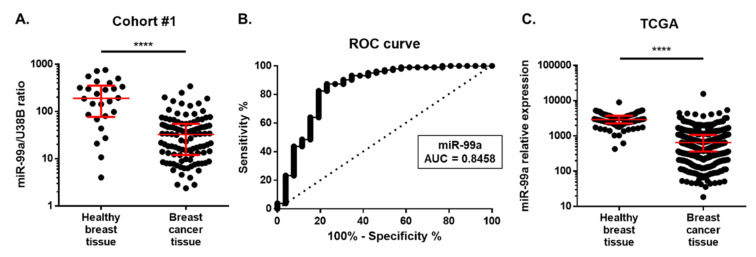
(**A**) MiR-99a expression levels in breast cancer tissues from Cohort #1. Differential miR-99a expression levels in 103 breast cancer tissues were compared with 26 normal breast tissues. Red horizontal line: median with interquartile range. Mann–Whitney U, **** *p* < 0.0001. (**B**) Receiver-operating characteristic (ROC) curve analysis for miR-99a expression levels in breast cancer tissue samples. (**C**) TCGA data for the expression of miR-99a-5p in normal solid tissue (*n* = 52) and breast primary tumour (*n* = 782). Expression is represented as reads per million miRNA mapped. Horizontal line: median with interquartile range. Mann–Whitney U, **** *p* < 0.0001.

MiR-99a-5p expression was determined in 103 BC tissues and 26 healthy breast tissues by qRT-PCR. Detailed clinical and pathological data are depicted in Table 1. Overall, the median age of controls was significantly lower than that of patients (*p* = 0.0228). Nonetheless, no correlation between miR-99a-5p levels and age was observed (data not shown). MiR-99a-5p expression levels were significantly lower in BC tissue (median, 95% CI: 32.72, 18.46–38.76) than in healthy breast tissues (median, 95% CI: 190, 85.20–317.80) (Figure 2A). MiR-99a-5p expression levels were able to discriminate BC from healthy breast tissues with an area under curve (AUC) of 0.8458 (95% CI: 0.7441–0.9474; *p* < 0.0001) (Figure 2B). Importantly, using the best cut-off value (78.17), 87.38% sensitivity, 76.92% specificity, and 85.27% accuracy were obtained. No significant associations were found between tissue miR-99a-5p levels and clinicopathological features (BC subtypes, histological grade, stage, pathological T stage, and regional lymph node metastasis) (Table 2).

Moreover, we also verified the expression of miR-99a-5p in The Cancer Genome Atlas (TCGA) cohort. Data from 52 normal solid tissues and 782 breast primary tumours confirmed that miR-99a-5p expression was higher in normal tissues than in tumour tissues (*p* < 0.0001) (Figure 2C).

### 2.3. MiR-99a-5p Expression in Plasma

#### 2.3.1. Cohort #2: Testing Cohort

**Figure 3:**
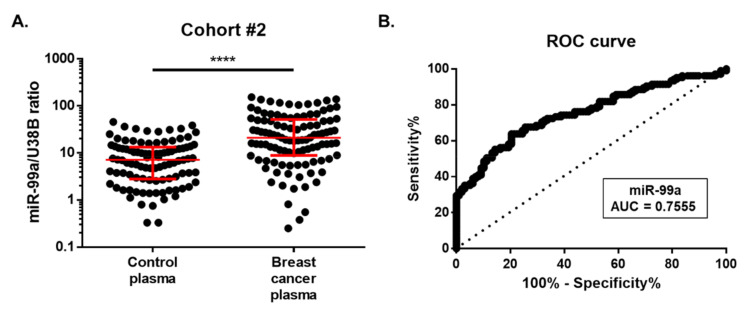
(**A**) Circulating miR-99a levels in cohort #2. Differential miR-99a levels in 105 plasma of breast cancer patients were compared with those of 98 healthy controls. Expression levels were significantly lower in healthy controls than in breast cancer patients. Red horizontal line: median with interquartile range. Mann–Whitney U, **** *p* < 0.0001. (**B**) Receiver-operating characteristic (ROC) curve analysis for circulating miR-99a levels in cohort #2.

Considering the promising results obtained in tissue samples, we proceeded to explore the diagnostic value of miR-99a-5p in liquid biopsies. Indeed, miR-99a-5p was evaluated in plasma samples from BC patients (*n* = 105) and healthy controls (*n* = 98) (cohort #2). Clinicopathological data are detailed in Table 3. No significant differences in median age between groups were observed. Herein, miR-99a-5p expression levels were significantly higher in plasma from BC patients (median, 95% CI: 21.02, 15.26 – 28.79) than in healthy volunteers (median, 95% CI: 7.09, 5.03 – 9.65) (*p* < 0.0001) (Figure 3A).

To assess the potential value of the miR-99a-5p expression in plasma for the diagnosis of BC, we computed the ROC curve for differentiating between BC patients and asymptomatic controls (Figure 3B**)**. The obtained AUC was 0.7555 (95% CI: 0.69–0.82; *p* < 0.0001). At the optimal cut-off value of 15.04, 63.81% sensitivity, 79.59% specificity, and 71.43% accuracy were obtained.

#### 2.3.2. Cohort #3: Validation Cohort

**Figure 4:**
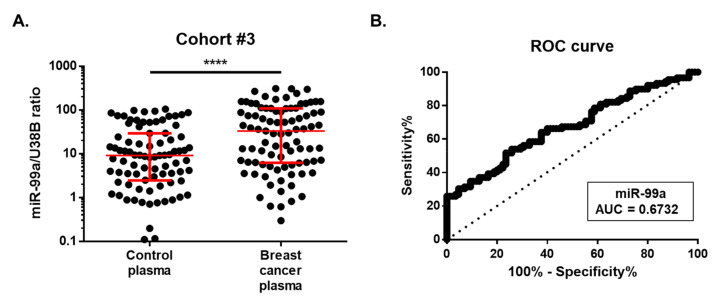
(**A**) Circulating miR-99a levels in cohort #3. Differential miR-99a levels in plasma of 89 breast cancer patients were compared with those of 85 healthy controls. Expression levels were significantly lower in healthy controls than in breast cancer patients. Red horizontal line: median with interquartile range. Mann–Whitney U, **** *p* < 0.0001. (**B**) Receiver-operating characteristic (ROC) curve analysis for circulating miR-99a levels in cohort #3.

The value of circulating miR-99a-5p levels as a BC biomarker was further assessed in an independent cohort (cohort #3: validation cohort) comprising plasmas of 89 BC patients and 85 asymptomatic controls (Table 3). No significant differences were observed between the median age of both groups. MiR-99a-5p levels were significantly overexpressed in the plasma of BC patients (median, 95% CI: 33.09, 12.95–54.40) than in controls (median, 95% CI: 9.11, 4.73–11.86) (*p* < 0.0001) (Figure 4A), in agreement with the results obtained for the testing cohort (cohort #2). Furthermore, using the cut-off value obtained from cohort #2 (15.04), miR-99a-5p was able to identify BC with 57.30% sensitivity, 67.06% specificity, and 62.07% accuracy. Nonetheless, a 0.6732 AUC (95% CI: 0.59–0.75; *p* < 0.0001) was observed for this set of samples (Figure 4B).

### 2.4. miR-99a-5p as a Biomarker for Early BC Detection

**Figure 5:**
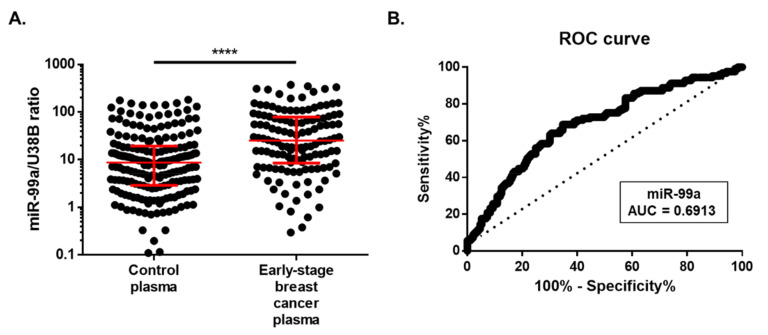
(**A**) Expression of miR-99a in early-stage breast cancer plasma. Distribution of circulating miR-99a levels in 125 plasma of early-stage breast cancer patients (stage I and II) and 193 healthy controls. Expression levels were significantly lower in healthy controls than in early-stage breast cancer patients. Horizontal line: median with interquartile range. Mann–Whitney U, **** *p* < 0.0001. (**B**) Receiver-operating characteristic (ROC) curve analysis for circulating miR-99a levels in early-stage breast cancer patients.

We further hypothesized that circulating miR-99a-5p might be used as a biomarker for non-invasive early BC detection. Patients from cohort #2 and cohort #3 were put together (194 plasma samples), as there were no statistical differences in miR-99a-5p expression between cohorts (*p* = 0.22, data not shown). No significant associations were found between circulating miR-99a-5p levels and clinicopathological features (BC subtypes, histological grade, stage, pathological T stage, regional lymph node metastasis, and distant metastasis) (Table 4). Interestingly, circulating miR-99a-5p levels were up-regulated in early BC patients (stage I and II) compared with asymptomatic controls (*p* < 0.0001) (Figure 5A). Moreover, using the optimal cut-off value of 12.75, circulating miR-99a-5p levels were able to discriminate early BC from healthy controls with a 66.67% accuracy, 68.80% sensitivity, 65.28% specificity, and an AUC of 0.6913 (95% CI: 0.63 – 0.75; *p* < 0.0001; Figure 5B).

Overall, these results provide evidence that circulating miR-99a-5p levels may be used to detect early BC patients.

## 3. Discussion

Breast cancer is the most common malignant tumour in the female population [1]. The importance of early detection of BC through the use of mammography and other techniques is fundamental as they change the prognosis of the disease. Therefore, the identification of biomarkers providing more accurate diagnostic information for BC patients is urgently needed.

In order to find new strategies for early diagnosis, several studies have been focused on miRNAs. These are a group of small non-coding RNAs that are involved in regulating a range of developmental and physiological processes, and their dysregulation has been associated with cancer. Specifically, circulating miRNAs have been proposed as being useful biomarkers for different cancer types’ detection. Indeed, miRNAs present advantages that make them interesting to be used as diagnostic tools. These include being stable molecules that can be easily detected in body fluids, such as plasma and the fact that their expression has been correlated with clinicopathological features, being promising as prognostic and predictive biomarkers [9].

Several authors assessed miR-99a expression in BC tissues and demonstrated its downregulation when compared to healthy tissues [23,24,25,26]. In this respect, miR-99a has been confirmed to be a tumour suppressor and its overexpression was associated with proliferation, invasion, and migration inhibition in BC cells *in vitro* and *in vivo* [23,27]. Moreover, that effect has been suggested to be mediated by several confirmed targets, such as mTOR [23,28], HOXA1 [24], IGF-1R [27], CDC25A [25], or FGFR3 [29]. Additionally, it is important to underline that miR-99a is significantly downregulated in several tumours, including glioma [30], oral squamous cell carcinoma [31], head and neck squamous cell carcinoma [22], endometrioid endometrial carcinoma [32], bladder cancer [21], non-small cell lung cancer [33], anaplastic thyroid cancer [34] or hepatocellular carcinoma [35]. All these findings suggest that miR-99a might be a potential cancer biomarker. In this study, we aimed to explore the value of circulating miR-99a-5p levels as a BC diagnostic biomarker.

Firstly, we evaluated miR-99a-5p expression in tissue samples from 26 healthy patients and 103 BC patients. The downregulation of miR-99a-5p in BC tissue was confirmed, in agreement with previous studies [23,24,25,26]. Besides, our result was also validated in the TCGA cohort.

Using plasma samples for diagnosis and follow-up of patients presents some advantages, as its extraction is less invasive than tissue biopsies. Many previous studies demonstrated the applicability of miRNAs in plasma as novel biomarkers for cancer diagnosis and prognosis [3,6,7]. Herein, we assessed circulating miR-99a-5p levels in two independent cohorts of BC patients and healthy volunteers from two different hospitals (testing and validation, respectively, cohorts # 2 and #3).

Surprisingly, contrary to the discovery cohort (cohort #1), in the testing cohort (cohort #2) from IPO-Porto, circulating miR-99a-5p expression levels were significantly higher in patients than in healthy controls. Then, our results were blindly validated in the validation cohort (cohort #3) from INCLIVA. Significant differences between groups were confirmed, suggesting that circulating miR-99a-5p concentrations may be a useful biomarker for the detection of BC.

Importantly, to validate the application of circulating miR-99a-5p levels as a diagnostic biomarker, optimal cut-off from cohort #2 was applied in cohort #3, and the value of circulating miR-99a-5p levels in discriminating BC patients from controls was confirmed. Moreover, we tested the capacity of miR-99a-5p to detect early BC (stages I and II), and our results suggest that circulating miR-99a-5p concentrations might accurately detect BC patients in early stages of the disease.

Although it was somewhat unexpected to find opposite trends in tissue and plasma samples, similar results have been already reported for this miRNA and others in different tumour models [36]. Specifically regarding miR-99a, Torres et al. found similar results in endometrial endometrioid cancer, miR-99a expression being downregulated in the tissue of patients compared with tissue controls, whereas it was overexpressed in patients’ plasma when compared to healthy samples [32,36]. Moreover, in the context of BC, several other microRNAs were demonstrated by other researchers to have opposite expression patterns in tissue and peripheral blood [19,20,37,38].

The processes by which miRNAs enter the bloodstream remain poorly understood. Nevertheless several reasons might underlie the differential circulating expression profiles found: (I) miRNAs might be released by cancerous cells in an active manner [39]; (II) miRNAs are passively released by apoptotic or necrotic cells [40]; (III) miRNAs may derive from tumour microenvironments [37]. Pigati et al. reported that released miRNAs may not, indeed, reflect the miRNA profile in the cell of origin, and demonstrated that the amount of released miRNA significantly differs between cell lines with similar death rates [39]. Hence, these results reinforce the concept of selective release, which may explain the expression profile’s differences also observed in the current study.

Intriguingly, several authors that determined miR-99a levels in serum samples from BC patients found opposite results compared to ours. First, Li et al. obtained serum from 72 BC patients and 40 healthy volunteers, and found that miR-99a-5p was downregulated in patients [41]. Then, Yu and co-workers determined miR-99a expression in serum samples of 113 BC patients and 47 healthy controls and found the same trend [42]. Nevertheless, several authors have already reported the lack of correlation between miRNAs expression in serum and plasma samples [43,44]. For instance, Li et al. found a negative correlation between plasma and serum of BC patients for some miRNAs [44]. The explanation for these findings might be that, before serum collection, blood is allowed to clot before obtaining the supernatant. Several authors consider that coagulation could cause cell lysis and blood cells would release miRNAs as a consequence, as well as loose of some vesicles that may be trapped in the coagulum [43,44]. Therefore, it is not possible to compare results from serum and plasma.

To our best knowledge, this is the first study to evaluate the value of circulating miR-99a-5p in plasma as BC detection biomarker. Our results provide the evidence to consider plasma miR-99a-5p as a non-invasive biomarker for early BC, which might contribute to improving early detection and consequently reduce BC-related mortality.

## 4. Materials and Methods

### 4.1. Clinical Samples

This retrospective study included non-consecutive female patients over the age of 18 years. Samples were collected from two independent institutions: Portuguese Oncology Institute of Porto (IPO-Porto, Portugal) and Biomedical Research Institute INCLIVA (Spain).

A discovery cohort included a total of 103 BC tissue samples available at the Biobank of the Department of Pathology from IPO-Porto (cohort #1). Furthermore, 26 healthy breast tissue samples collected from reduction mammoplasties of the contralateral breast from BC patients were also included for our purposes. All these specimens were obtained from patients without BC hereditary syndrome and no evidence of preneoplastic/neoplastic lesions. After surgical resection, samples were immediately frozen at −80 °C. Five micrometer frozen sections were cut and stained with hematoxylin-eosin for confirmation of BC by an experienced pathologist, ensuring that samples contained at least 70% of tumour cells, and confirming that tissues obtained from reduction mammoplasties harboured normal epithelial cells.

The testing cohort included a total of 105 patients with BC diagnosed at the IPO-Porto, for which plasma samples were available (cohort # 2). All samples were collected before any treatment. For control purposes, plasma samples were collected from 98 healthy donors from the same institution. After the collection of peripheral blood into EDTA-containing tubes, plasma was obtained by centrifugation at 2000 rpm for 10 min at 4 °C and was stored at -80 °C until further use.

The validation cohort (cohort #3), included 89 BC patients from INCLIVA with plasma samples collected before treatment, and control plasma samples collected from 85 healthy donors from the same institution and Valencian Biobanking Network. After the collection of peripheral blood into EDTA-containing tubes, plasma was obtained and stored as described for cohort #2.

This study was approved by the institutional ethical committees of IPO-Porto (CES-IPOFG-120/015) and INCLIVA (2019/196). Informed consent was obtained from all patients and donors included in the study. Sample collection was performed in accordance with the Declaration of Helsinki.

### 4.2. RNA Extraction from Tissue and Plasma

Total RNA from tissue using TRIzol^®^ Reagent (Invitrogen, Carlsbad, CA, USA) according to the manufacturer’s recommendations. RNA concentrations and purity ratios were determined using a NanoDrop Lite spectrophotometer (NanoDrop Technologies, Wilmington, DE, USA). MiRNAs were extracted from plasma samples using miRNeasy Serum/Plasma Kit (Qiagen, Hilden, Germany), according to manufacturer’s instructions. RNA samples were stored at −80°C.

### 4.3. cDNA Synthesis

For cDNA synthesis, 500 ng of total RNA from tissue, or 9.16 µL of miRNA from plasma were used. TaqMan™ MicroRNA Reverse Transcription Kit (Thermo Fisher Scientific, Waltham, Massachusetts, USA) was employed, according to the manufacturer’s protocol in a total volume of 15 µL. For the synthesis of cDNA, reaction mixtures were incubated in a thermal cycler at 16 °C for 30 min, at 42 °C for 30 min, and at 85 °C for 5 min.

### 4.4. miRNA Expression Analysis

Expression levels of miR-99a-5p (Assay ID 000435) and the reference gene RNU38B (Assay ID 001004) were analysed in triplicate via quantitative real-time PCR (qRT-PCR) using the human TaqMan microRNA Assay kit (Thermo Fisher Scientific Waltham, Massachusetts, USA). Then, 2 µL of cDNA solution were amplified with 5 µL of Xpert Fast Probe 2x MasterMix (GRiSP, Portugal), 0.5 μL of gene-specific primers/probe, and 2.5 µL of nuclease-free water in a final volume of 10 µL. qRT-PCR of cohort #1 and cohort #2 were run in a LightCycler 480 Instrument (Roche Diagnostics, Manheim, Germany), and cohort #3 was run in a QuantStudio 5 Real-Time PCR System (Thermo Fisher Scientific Waltham, Massachusetts, USA). Reaction mixtures were incubated at 98 °C for 3 min, followed by 45 cycles of 95 °C for 10 s, 60 °C for 30 s, and 37 °C for 30 s. Five serial 10X dilutions of positive control were run in each plate to generate a standard curve, which was used to calculate the expression level of miRNAs.

### 4.5. TCGA Database Validation

The expression levels of miR-99a-5p were validated in the TCGA dataset. miRNA expression data were downloaded from OncoMir Cancer Database (OMCD) (https://www.oncomir.umn.edu/omcd/basic_search.php).

### 4.6. Statistical Analysis

To evaluate differences in the miRNA expression levels and associations between miRNA expression and clinical variables, Mann–Whitney U and Kruskal–Wallis tests were used. Receiver-operating characteristic (ROC) curves were constructed by plotting the true positive (sensitivity) against the false-positive (1-specificity) rate, and AUC was calculated. Optimal cut-off values were established based on the highest value obtained in ROC curve analysis according to Youden’s J index [45,46]. Then, specificity, sensitivity, and accuracy were determined. In cohort #3 (validation cohort), specificity, sensitivity, and accuracy were determined by applying cut-off obtained in cohort #2 (testing cohort). Statistical analyses were performed using GraphPad Prism 6.01 software for Windows (GraphPad Software, La Jolla, California, USA). Results were considered statistically significant when the *p*-value was <0.05.

## 5. Conclusions

Overall, our results show that circulating miR-99a-5p levels in plasma, might be clinically useful as a non-invasive BC cancer detection biomarker, namely in the early stages of the disease. Importantly, similar results were obtained in two independent cohorts of BC patients from two different hospitals. Nonetheless, validation in larger multi-institutional cohorts are necessary to confirm these results.

## Figures and Tables

**Table 1:** Clinicopathological characteristics of breast cancer patients and controls in discovery cohort: cohort #1.

Characteristics		Tissue Samples	
		Breast Cancer Patients	Controls
Number		103	26
Median age, years (range)		59.7 (57–62)	54.6 (47–63)
Molecular subtype, *n* (%)			
	Luminal	59 (57.3%)	n.a.
	TNBC	30 (29.1%)	
	Her 2	14 (13.6%)	
Grade group, *n* (%)			
	1	9 (8.7%)	n.a.
	2	36 (35%)	
	3	46 (44.7%)	
	Unknown	12 (11.6%)	
Stage, *n* (%)			
	I	12 (11.7%)	n.a.
	II	63 (61.2%)	
	III	13 (12.6%)	
	Unknown	15 (14.6%)	
Pathological T stage, *n* (%)			
	pT1	24 (23.3%)	n.a.
	pT2	57 (55.3%)	
	pT3	6 (5.8%)	
	pT4	1 (1%)	
	Unknown	15 (14.6%)	
Regional lymph node metastasis, *n* (%)			
	No	39 (37.9%)	n.a.
	Yes	50 (48.5%)	
	Unknown	14 (13.6)	
Distant metastasis, *n* (%)			
	No	89 (86.4%)	n.a.
	Yes	0 (0%)	
	Unknown	14 (13.6%)	
TNBC, triple-negative breast cancer; n.a., not applicable			

**Table 2:** Association between tissue miR-99a levels and clinicopathological features of breast cancer patients (cohort #1).

		Number (%)	Median (95% CI)	*p* Value
Histological subtype, *n* (%)				
	Luminal	59 (57.3%)	27.00 (31.25–55.26)	0.9783
	TNBC	30 (29.1%)	33.65 (25.62–44.83)	
	Her 2-enriched	14 (13.6%)	18.51 (8.64–93.79)	
	Unknown	n.a.		
Grade group, *n* (%)				
	1	9 (8.7%)	38.76 (9.45–110.10)	0.7869
	2	36 (35.0%)	18.18 (25.27–56.10)	
	3	46 (44.7%)	32.33 (25.31–57.65)	
	Unknown	12 (11.6%)		
Stage				
	Early (I and II)	75 (72.8%)	21.38 (29.78–54.31)	0.8250
	Late (III and IV)	13 (12.6%)	35.87 (19.85–43.36)	
	Unknown	15 (14.6%)		
Pathological T stage, *n* (%)				
	pT1	24 (23.3%)	23.40 (18.43–52.7)	0.687
	pT2	57 (55.3%)	23.05 (28.32–48.89)	
	pT3	6 (5.8%)	24.57 (-61.25–213.1)	
	pT4	1 (1%)	54.97	
	Unknown	15 (14.6%)		
Regional lymph node metastasis, *n* (%)				
	No	39 (37.9%)	18.51 (23.08–64.21)	0.6279
	Yes	50 (48.5%)	33.90 (28.52–49.03)	
	Unknown	14 (13.6)		
TNBC, triple-negative breast cancer; n.a., not applicable				

**Table 3:** Clinicopathological characteristics of breast cancer patients and controls of testing and validation cohorts: cohort #2 and cohort #3.

	Plasma Samples			
	Cohort #2		Cohort #3	
	Breast Cancer Patients	Controls	Breast Cancer Patients	Controls
Number	105	98	89	85
Median age, years (range)	52 (29–82)	50 (40–64)	54.1(32–92)	55 (32–90)
Histological subtype, *n* (%)				
Luminal	92 (87.6%)	n.a.	54 (60.7%)	n.a.
TNBC	7 (6.7%)		15 (16.9%)	
Her 2-enriched	5 (4.8%)		18 (20.2%)	
Unknown	1 (1%)		2 (2.2%)	
Grade group, n (%)				
1	8 (7.6%)	n.a.	19 (21.3%)	n.a.
2	54 (51.4%)		44 (49.4%)	
3	39 (37.1%)		25 (28.1%)	
Unknown	4 (3.8%)		1 (1.1%)	
Stage, *n* (%)				
I	42 (40%)	n.a.	24 (27.0%)	n.a.
II	18 (17.1%)		41 (46.1%)	
III	32 (30.5%)		15 (16.3%)	
IV	13 (12.4%)		5 (5.4%)	
Unknown			4 (4.3%)	
Pathological T stage, *n* (%)				
pT1	46 (43.8%)	n.a.	36 (40.4%)	n.a.
pT2	29 (27.6%)		37 (41.6%)	
pT3	18 (17.1%)		9 (10.1%)	
pT4	10 (9.5%)		1 (1.1%)	
Unknown	2 (1.9%)		6 (6.7%)	
Regional lymph node metastasis, *n* (%)				
No	53 (50.5%)	n.a.	47 (52.8%)	n.a.
Yes	50 (47.6%)		36 (40.4%)	
Unknown	2 (1.9%)		6 (6.7%)	
Distant metastasis, *n* (%)				
No	92 (87.6%)	n.a.	80 (89.9%)	n.a.
Yes	13 (12.4%)		7 (7.9%)	
Unknown			2 (2.2%)	
TNBC, triple-negative breast cancer; n.a., not applicable				

**Table 4:** Association between circulating miR-99a levels and clinicopathological features of breast cancer patients (cohorts #2 and #3).

		Number (%)	Median (95% CI)	*p* Value
Histological subtype, *n* (%)				
	Luminal	146 (75.3%)	24.42 (16.00–36.14)	0.0590
	TNBC	22 (11.3%)	9.50 (3.46–33.22)	
	Her 2-enriched	23 (11.9%)	29.93 (13.53–89.41)	
	Unknown	3 (1.5%)		
Grade group, *n* (%)				
	1	27 (13.9%)	14.86 (5.61–36.9)	0.4594
	2	98 (50.5%)	28.29 (18.05–44.09)	
	3	64 (33.0%)	18.06 (12.88–36.14)	
	Unknown	5 (2.6%)		
Stage				
	Early (I and II)	125 (64.4%)	24.26 (15.98–36.90)	0.2382
	Late (III and IV)	65 (33.5%)	21.02 (12.08–35.81)	
	Unknown	4 (2.1%)		
Pathological T stage, *n* (%)				
	pT1	82 (42.3%)	24.23 (15.98–38.61)	0.4119
	pT2	66 (34.0%)	27.26 (12.91–44.91)	
	pT3	27 (13.9%)	10.99 (3.54–49.49)	
	pT4	11 (5.6%)	35.81 (5.34–48.45)	
	Unknown	8 (4.1%)		
Regional lymph node metastasis, *n* (%)				
	No	100 (51.5%)	24.23 (15.98–38.61)	0.3232
	Yes	86 (44.3%)	19.54 (12.91–35.84)	
	Unknown	8 (4.1%)		
Distant metastasis, *n* (%)				
	No	172 (88.7%)	20.03 (15.18–29.70)	0.1810
	Yes	20 (10.3%)	35.82 (16.2–91.99)	
	Unknown	2 (1.0%)		
TNBC, triple-negative breast cancer; n.a., not applicable				
